# Virtual Reality and the Mediation of Acute and Chronic Pain in Adult and Pediatric Populations: Research Developments

**DOI:** 10.3389/fpain.2022.840921

**Published:** 2022-05-06

**Authors:** Yacine Hadjiat, Serge Marchand

**Affiliations:** ^1^Department of Surgery, Service of Neurosurgery, French National Institute of Health and Medical Research (Inserm) U987, Paris-Saclay University (EDSP), Paris, France; ^2^Inserm U987, Pharmacology & Physiopathology, University de Sherbrooke University Hospital, Quebec, QC, Canada

**Keywords:** virtual reality, pain, distraction, analgesia, complementary pain management, acute pain, chronic pain, digital therapeutics

## Abstract

The use of virtual reality (VR) in the mediation of acute pain in adults has shown real benefit to patients for the past 20 years. This review of the literature provides a descriptive synthesis of the types of VR technology, the mechanisms by which VR mediates pain, and a history of early research in the area. A review of the use of VR to mediate chronic pain in adults, and both acute and chronic pain in pediatric populations follows. The studies reviewed provide mixed results and it is noted that many studies have small sample sizes, are case studies, and do not control for extraneous variables such as the dosage and type of VR technology used. Although VR is an exciting area of inquiry that promises to yield multiple applications, there is a necessity to conduct larger random controlled trials to better understand the use cases for which VR is most effective.

## Introduction

The control and treatment of pain remains a topic of great interest and importance. While the use of pharmacology is undoubtedly the most effective and efficient way to treat pain, there is growing concern about the side effects of traditional pain medications, such as opioids, and the development of tolerance to pain medications. In addition, pain is a subjective experience that is influenced by a plethora of factors that are not physical in nature. Social and cultural factors, as well as gender and personal parameters, impact the experience of pain (1). There is also a proven link between acute pain and anxiety, depression and other mental health conditions. Chronic pain and anxiety disorders frequently co-exist and exacerbate each other, and depression and anxiety are associated with increased perception of pain severity (1).

Adjunctive non-pharmacologic techniques, such as hypnosis, cognitive behavioral therapy and music have been used to complement the pharmacological treatment of procedural pain, but the magnitude of these benefits has only been partially embraced by mainstream science (2–4). Physicians are now increasingly investigating complementary and non-traditional ways to manage pain, including the use of virtual reality (VR). VR is a multi-sensory technology that has been used in clinical settings for over 20 years. It is characterized by its non-pharmaceutical interventions that have been shown to play a role in pain modulation by distraction or VR analgesia (5), and its ability to enable emotional processing and reduce treatment avoidance (5).

Without significant clinical research on efficacy, VR and especially Head Mounted Device (HMD) VR, may not be considered a cost-effective complementary pain intervention. Research on both the experience of pain and the associated negative emotions of being exposed to a painful medical procedure are necessary to support the value of VR. If the use of VR is proven to be effective, the benefits could extend beyond pain reduction. The use of VR to complement and enhance pain relief could lead to reduced use of pharmaceutical analgesia and therefore exposure to its side effects and risks (6).

A number of systematic reviews and meta-analyses on specific aspects of the use of VR and pain have been conducted. For example, Chan, Foster, Sambell and Leong conducted a review of the use of VR in acute procedural pain management (7); and Ding et al. (8) conducted a systematic review and meta-analysis of the effects of VR in relieving post-operative pain in surgical patients. The current review differs from these analyses by providing a more holistic overview of the history of VR in managing pain, the mechanisms of VR analgesia, and the use of VR in mediating acute and chronic pain in adult and pediatric populations.

The objectives of the review are to provide an introduction and overview of research related to the use of VR to manage pain, providing greater breadth to reviews previously undertaken. The focus on breadth, as opposed to depth in one specific setting, aims to stimulate interest and research in the field by exposing readers to the numerous applications and settings in which VR can be used to manage pain.

## Methodology

Google Scholar was used to search for relevant studies. Searches were performed between 22 September 2021 and 30 January 2022 using a combination of medical subject headings and free-text terms. After preliminary searches, the following combined terms were used: “Virtual reality AND distraction,” “Virtual reality AND pain,” “Virtual reality AND analgesia.” The terms “acute,” “chronic,” “pediatric,” and “immersive” were then added. A hand search of reference lists of included studies and previously published systematic reviews was also conducted.

A meta-analysis could not be conducted due to differences in study designs and types of controls. A descriptive synthesis is presented.

## VR Technology Options

Virtual reality is not a standard intervention and there are numerous ways in which the technology itself can be applied. It can be used to create a fully or partially virtual world (9). Mazurek et al. describe four categories of VR starting with reality/the real world. The second category is augmented reality where computer-generated data merge into a real-world image. The third category is augmented virtuality where real-life data are merged into a computer-generated world. The fourth category is VR where the world is created entirely by a computer (10). In order to create a fully virtual world, all senses need to be included – sight, sound, smell, taste, and touch (10).

A further distinction in VR technology is whether the experience is immersive or non-immersive. VR is considered immersive when a head-mounted display (HMD) is used. When the virtual environment is presented on a flat screen (e.g., computer screen) the experience is considered non-immersive (11). When using immersive VR systems, the patient/subject is embodied in a virtual body and immersed in the virtual world. This causes them to feel “present” in the generated VR scenario and this sense of presence leads to the reduction of sensations of pain (12).

Some studies have found Immersive VR to be more effective in promoting analgesia than non-immersive VR systems (13–17). Austin et al. compared the use of 3D HMD (immersive) and 2D screen sessions in people with neuropathic Spinal Cord Injury pain. They found significant decreases in pain intensity during and immediately after taking part in both 3D HMD and 2D screen sessions, but the reduction in pain intensity was significantly greater with 3D HMD VR (18).

Others have found that the success of VR is not dependent on having an HMD (19). Mott et al. showed significant results using augmented reality (where VR is overlaid on the material world) (19). However, Li et al. (20) do raise the point that high quality VR has shown a significant positive effect over low quality VR. Another study used a novel robot-arm like device for the VR system to avoid face and head discomfort for patients with severe burns, and results demonstrated the positive impact of VR vs. non-VR in a randomized control experiment of 12 people (16).

Within immersive VR systems, it is now possible to create interactive experiences. Portable and affordable motion tracking systems allow for movements of a virtual body, called an avatar, to be controlled by movements of the user's real body (21). Even more advanced technology has been developed where infrared cameras and sensors attached to participants' bodies are now being used (22).

Interactive VR was used in a randomized controlled trial of 48 healthy volunteers to investigate the intensity or level of distractibility of the VR technology used. Two VR conditions were compared – interactive VR using eye-tracking integrated into the helmet and passive/no eye-tracking VR. Each participant received two nociceptive thermal stimuli – one during the active VR condition and one during the passive VR condition (order randomized). Active VR decreased the ratings of pain unpleasantness and pain severity. The subjects also reported higher levels of feeling “present” and greater immersion, supporting the theory of attention distraction in pain relief (23).

## Mechanisms of VR Analgesia

Pain is a complex and dynamic phenomenon. From the nociceptive stimulation to the perception of pain, several endogenous mechanisms can increase or decrease the signal, changing the pain experience. The final experience of pain implies a sensory activation to identify location, intensity or duration of the nociceptive activity. This sensory component of pain is generally related to activities in brain structures such as the somatosensory cortices S1 and S2 (24). But there is also an important affective component that will trigger different emotions related to the unpleasantness of the experience. The affective component of pain is related to the activity of regions such as the cingulate cortex, the insula and the prefrontal cortex (24). The affective component of pain seems to play an important role in the amplification and persistence of certain pain conditions and can even produce a “genomic” memory of pain in the adult cortex (25).

Cognitive and affective components can have a positive effect in the treatment of pain. Bushnell et al. assert that life-style choices or mind-body techniques, such as the practice of meditation or yoga, can reduce pain perception (26). Meditation and yoga have been found to decrease both the sensory-discriminative and affective-motivational aspects of pain even while not actively engaged in the practice (26). Social support has also been associated with lower levels of chronic pain, labor pain, cardiac pain, postoperative pain, and the reduction of perception of induced pain in controlled experimental settings (27). VR analgesia most likely draws on these cognitive and affective endogenous pain modulation mechanisms. Considering the importance of the immersive aspect of VR, distraction has been suggested to be the main mechanism of VR pain reduction (28–31).

Ahmadpour et al. explain the construct of distraction in relation to VR (32). They describe people feeling a sense of “presence” while in immersive virtual environments, feeling as though one is actually there. When the nature of the immersion is strong and effective, the person feels that they inhabit the virtual world and not the real world where the pain is being experienced. In these circumstances, the person's ability to respond to noxiouis stimuli and attend to nociceptive neural signals is compromised, leading to them perceiving less pain. This mechanism is described as distraction (32, 33).

Melzack's Neuromatrix Theory of Pain supports the theory of distraction, asserting that inputs such as cognition, sensation and affect can alter pain output. Not only is this mechanism linked to the perception of pain, it differs from the mechanism used by many analgesics that disrupt pathways that transmit nociceptive signals to the central nervous system (32, 33). Because of its central protective role, pain is intrinsically salient, however, if an important distracting event happens concomitantly, the salience of pain can be reduced to the point of being ignored (28). Interacting with virtual reality causes patients to divide their attention and they thus have less attention available to process incoming signals from pain receptors. The result is that patients spend less time thinking about pain and experience less pain when using VR (4).

Virtual reality adds the dimension of immersion and heightened distraction by appealing to many senses and gaining more of the patients' attention. Virtual environments use head tracking systems, tactile feedback and highly stimulating visual and auditory sensations. In this way, the individual becomes immersed in the virtual world, taking attention away from the perception of pain (5). Mechanisms other than distraction may also play a role. fMRI studies have shown that patients using VR have reduced signaling in the cortical regions of the pain matrix (much like other pain reduction methods). Other cortical areas are undoubtedly also at play as reduction in pain is also associated with attention distraction, task loading, moods, expectancy and perceived controllability. Areas such as the cingulate gyrus, insula, thalamus and primary somatosensory cortex have all been implicated in pain responses, although some regions are more related to specific sensory and affectives aspects of pain it is not yet clear which areas are responsible for the perception of pain intensity and which are associated with the affective component of pain – all of which would need to be clearly delineated in research (20, 34).

Gold et al. (29) explore the role of the underlying neurobiology of VR pain attenuation. They explain that analgesia can be achieved by interrupting the body's normal means of detecting pain. This is traditionally achieved by targeting C fibers, a type of unmyelinated neuron responsible for dull and aching chronic pain as well as the secondary pain associated with acute pain. Other therapies act within the brain to produce analgesia. They posit that VR may also act within the brain, but by acting directly and indirectly on pain perception and signaling through attention, emotion, concentration, memory, and other senses (e.g., touch, auditory, visual), VR may change the activity of the body's intricate pain modulation system, thus altering pain perception (p.539) (29).

Recent studies suggest that VR pain reduction is at least partially triggered by descending pain inhibitory mechanisms originally known as Diffuse Noxious Inhibitory Mechanisms from the Le Bars animal studies (35, 36) and renamed Conditioned Pain Modulation (CPM) for human research. DNIC or CPM is based on the principle of pain being reduced by a concomitant painful stimulation - “pain inhibits pain” or “counterirritation” (37). Le Bars (1979) proposed that a nociceptive stimulus sends afferences to the periaqueducal gray matter (PAG) and Nucleus Raphe Magnus (NRM) of the brainstem, recruiting inhibitory output at multiple levels of the spinal cord. Animal studies demonstrate that a lesion of the dorsolateral funiculus, the main descending inhibitory pathway, will produce hyperalgesia, suggesting the existence of a tonic descending inhibition under normal conditions (38, 39). Moreover, studies suggests that some chronic pain conditions are related to a deficit of CPM (40–42).

Studies comparing CPM and distraction in the same subjects suggest different mechanisms and possible additive effects (43). Moreover, CPM can be measured after the conditioning stimulation, reducing the distracting effect of a concomitant stimulation (44).

It is believed that attention, emotion and memory can play a significant role in sensory perception, and that they affect the degree to which pain sensation enters an individual's awareness (29). Mayer et al. (43) described a descending pain-control system originating in the brain. When this pain-control system was activated *via* the fibers descending from the periaqueductal gray (PAG) area of the midbrain, analgesia was achieved. “The discovery that the PAG receives inputs from a variety of areas of the brain, notably cortical regions involved in attention and emotion, suggests that modulation of the descending pain-control system may underlie the profound effects of attention and emotion” (28). Interestingly, suggestions can significantly modulate the efficacy of Conditioned Pain Modulation and this effect is also recorded at the spinal level (45). Moreover, we can trigger a CPM by asking the subject to watch videos of a loved-one or oneself during a cold pressor test used to trigger CPM (46). These results emphasize the importance of cognitive and emotional component such as empathy on pain perception and physiological responses.

The anterior cingulate cortex (ACC) is another region of the brain that is important in understanding the way in which VR may mediate pain perception. Bantick et al. assert that the perigenual ACC mediates attentional processes and emotional reactions to pain (47). This finding is supported by deCharms et al. (37) who demonstrated that activation of the perigenual cingulate (using fMRI feedback to control this brain activity) produced analgesia.

Gold et al. (29) hypothesize that the ACC exerts effects on structures such as the PAG, and that this is a critical component of the VR mediated pain-modulation pathway. VR is thus thought to mediate pain by activating the perigenual ACC, which in turn activates the PAG, initiating a cascade of signaling events to stimulate the descending pain-modulation system and produce analgesia. Although much research has been conducted, Gold et al. (29) state that the “neurobiological mechanisms underlying VR's action remain enigma” (p.541) and conclude that further research is required.

Research has also found specific autonomic responses when comparing VR and a 2D distraction video of the same scene. The authors showed that autonomic responses recorded by heart rate variability and galvanic skin responses had significantly higher parasympathetic activity during the VR session, suggesting a release of the tonic sympathetic activity normally recorded during thermal stimuli as used in this project (48). Interestingly, descending pain inhibitory mechanisms (CPM) are suggested to be related to autonomic responses (49, 50).

Finally, recent studies with chronic pain patients suggest that there may be an additive effect of the repetitive use of VR (51). The above research suggests that there is more than merely a distraction effect involved in VR pain control. Better understanding of these mechanisms will help direct future treatment development. In addition, personalization may play a role.

## Pioneers in Virtual Reality and Acute Pain in Adults

Hunter Hoffman and David Patterson are recognized as pain specialists and VR pioneers. They are both researchers at the University of Washington and have worked for over 20 years to demonstrate VR's unique ability to alleviate acute pain. Their seminal work was conducted with burn patients. They developed a VR game called SnowWorld where patients use wide-view goggles, an audio headphone, and a simple hand controller to interact with snowmen, igloos, penguins, wooly mammoths, and flying fish by throwing snowballs (4, 52). Immersing themselves in SnowWorld and focusing on the game during treatments, led burn patients to report 35–50% reductions in procedural pain. fMRI brain scans also showed associated reductions in pain-related brain activity during VR (4).

Hoffman et al. (53) then conducted research in healthy subjects, exposing them to thermal stimulation. They found that VR significantly reduced pain and they identified five brain regions of interest - the anterior cingulate cortex, primary and secondary somatosensory cortex, insula, and thalamus (53). Hoffman and his colleagues have presented numerous studies of pain reduction using VR, with reported reductions in pain intensity, anxiety and attention to pain (5, 54). They also conducted a randomized control study (RCS) with results supporting the case study observations (55). In the RCS, Hoffman et al. investigated pain intensity in burn patients undertaking range of motion physical therapy. There was a significantly lower rating of pain between an analgesia-only group and an analgesia-plus-VR group; with the latter group also showing greater range of motion. fMRI scans showed that the pain reduction experienced by using VR was comparable to the analgesic effect of a moderate dose of hydromorphone pain medication (55).

The majority of early studies on VR and pain targeted burn patients as participants due to the levels of pain, discomfort and anxiety experienced (16, 20). These studies focused on the physical sensation and reporting of pain, with a few also indicating the emotional states associated with pain (5). Later studies have found positive results in pain reduction in different settings including pain from chemotherapy port access (20), lumbar punctures (56), gastrointestinal endoscopy (57), rehabilitation of upper limbs of patients with Duchenne muscular dystrophy (58), headaches and fibromyalgia (31). Later studies have also included a broader focus on emotional states such as anxiety, fatigue, and depression (52).

Not all early research found a positive relationship between the use of VR and the mediation of pain. Mahrer and Gold provide a review of early research into VR and pain analgesia, finding differing results across studies (5). Some demonstrated equal efficacy for distraction and VR conditions (59), while others found no self-reported differences in pain perception (60). In the latter case, care givers did, however, note a reduction in distress behaviors.

## The Use of VR in Managing Chronic Pain in Adult Populations

By far the majority of research on pain and VR has been in the area of acute pain or pain associated with procedures. Additional research is needed to understand the role of VR in managing chronic pain. The small number of studies completed indicate positive potential. Sarig-Bahat et al. used VR to promote range of movement in people with neck pain and found a significantly reduced level of pain (61).

A study of 30 patients with chronic pain from many conditions (and therefore not controlling for pain type or location) was conducted where participants were provided with a fantasy world immersive VR session of 5 min. There was a statistically significant effect of pain reduction in the VR conditions. Pain was reported to be reduced by 60% between the pre-VR vs. during the VR session. The pain decreased by 33% between the pre-VR condition and the post-VR condition, demonstrating the lasting impact of the effect (62).

A systematic review and meta-analysis of VR as an analgesic for acute and chronic pain in adults by Mallari et al. suggests that the results of studies exploring the use of VR in mediating chronic pain are not consistent. They speculate that VR may effectively reduce chronic pain intensity during and possibly immediately after the VR exposure. However, they do not believe there is likely to be a lasting analgesic effect in patients with chronic pain conditions with the treatment protocols tested to date (63). They propose that the inconsistency in results may be caused by a variance in dosage of VR (the time a patient is exposed to VR) and the type of VR equipment used (63). The need for high quality randomized clinical trials for VR in patients with chronic pain conditions is thus required before definitive conclusions can be drawn.

Recent studies using a home-based VR treatment over a period of 8 weeks found a significant effect for VR compared to a sham VR (63). These results are encouraging, but more research over longer periods of time is required, as is the measurement of long post-VR effects to verify the lasting effect of a treatment.

When considering the use of VR for chronic pain, the question of whether one can become habituated to VR has been raised, but research does not support this. Rutter et al. found that over eight sessions of VR the significant decrease in pain intensity, anxiety and thoughts of pain remained across the administrations of a cold pressor (64). And earlier, Hoffman et al. (53) demonstrated significant reductions in pain over multiple physical therapy sessions for a burn patient, where the effect was retained regardless of the treatment duration and repeated treatment conditions.

## Research in Healthy Adult Populations

The impact of VR on pain tolerance and pain threshold has been further investigated in healthy populations, demonstrating a significant effect in increasing the threshold and tolerance levels (65). In these instances, factors such as cause of pain, environment, levels of anxiety and so forth can be controlled and address the challenges of the majority of existing research that is not completely generalizable. Li et al. summarizes studies where pain was created using a tourniquet, blood pressure cuff, cold pressor, and thermal pain stimulation. All studies found VR reduced pain intensity and the emotional distress that accompanies pain (20).

Another study in a healthy population was conducted by Loreto-Quijada et al. (66) in order to investigate the positive effects of VR on experimental pain. Seventy-seven students participated in a cold pressor pain test and were divided into two groups. One group's VR was intended to be a distraction from pain and the other VR group was intended to enhance pain control. Measures of self-efficacy, catastrophizing, pain intensity, tolerance, threshold, perception of time, and pain sensitivity were collected. The VR distraction condition was better than the control group for pain tolerance and pain threshold, but there was no significant difference for the cognitive aspects (66).

Hoffman et al. (53) investigated the neural correlates of VR pain attenuation by exposing study participants to periods of VR or no VR while enduring experimentally induced thermal pain while in a functional MRI (fMRI). VR not only significantly reduced subjective pain scores but also diminished pain-related brain activity in five regions associated with pain sensation. Several additional studies of clinically healthy subjects undergoing experimentally induced ischemia have demonstrated that VR can increase pain tolerance (32, 65), decrease self-reported pain intensity (32, 67), and reduce the affective unpleasantness and time spent thinking about pain (32).

Research in healthy populations allows for the control of numerous variables, but whether this research can be extrapolated to patients who are suffering from clinical pain that is beyond their control, is unclear.

## The Use of Virtual Reality to Manage Pain in Pediatric Populations

The question of whether VR can be successfully used to mediate pain in pediatric populations has been posed by many researchers. A large random controlled trial (RCT) by Sharar et al. (55) found significant effects for VR that were not influenced by age. Yet smaller studies do point to certain differences in age groups, often linked to efficacy and the type of VR used. In addition, there have been different results based on the procedure. There is no universally applied definition of what constitutes a pediatric population in the studies included in the current review. Age ranges start from 4 years old and extend to 21 years old.

Aminabadi et al. (68) found a positive impact of VR in children as young as 4–6 years old in dental conditions. Gold et al. replicated the significant lowering of pain in milder pain cases (blood draw in children 8–12 years) where a VR HMD resulted in lower pain compared to a no distraction condition, VR from a computer, and cartoons. Importantly in all conditions, the children could not see the procedure, therefore controlling for visual perception as a mediating factor affecting perception of pain. While the VR conditions did show a significant decrease in pain perception, the VR was associated with increased state anxiety (69).

In 2018 Gold and Mahrer undertook a randomized control study of 143 patients having blood drawn. The age range was between 10 and 21 years, and findings demonstrated lowered levels of pain for the VR condition and lowered levels of anxiety, with the effect of lower anxiety most prominent in those with reported high anxiety (70). Dahlquist et al. (71) found that in a group of children 6–14 years, VR with HMD helped the older children, but did not impact the younger ones. Age cohorts therefore need to be taken into consideration and findings cannot be extrapolated to all pediatric age ranges.

Additional randomized studies have demonstrated the effectiveness of VR in pain management in a variety of medical circumstances, including adolescents with cancer undergoing lumbar puncture (72), pediatric oncology patients requiring invasive medical procedures and chemotherapy (73, 74), children receiving presurgical anesthesia (75), children undergoing routine outpatient venipuncture (76), IV placement (77), and pediatric patients undergoing venipuncture and wound care in the emergency department (78). All of these research studies conclude that VR is effective in mediating pain in these pediatric populations.

Iannicelli et al. (79) published the first systematic review of VR compared to standard care in the pediatric population. They note a dearth in the literature on standardized and specific research using VR in pediatric populations. In the research that they included in their review, they found that despite the use of various methods to carry out the studies, VR distraction showed a statistically significant reduction in pain. The samples of subjects were between 30 and 252 subjects, while the age range was between 4 and 20 years (79). They state that all studies were conducted on acute pain and that further research is required to investigate the use of VR in mediating chronic pain in pediatric populations (79).

A recent (2020) review of 17 RCTs investigating VR distraction for acute pain in children included 1,008 participants aged 4–18 years, undergoing various procedures in healthcare settings (80). Again no meta analysis was possible due to the varied ages, procedures and settings. A narrative description of results reveals most studies to be at unclear risk of selection bias, high risk of performance and detection bias, and high risk of bias for small sample sizes. In addition, the authors downgraded the certainty of evidence to low or very low due to serious study limitations and imprecision. Within this context, the authors conclude that it is difficult to interpret the benefits of VR distraction for acute pain in children (81).

Research results are thus mixed and while use of VR for mediating acute pain in pediatric settings has yielded some positive findings, it is clear that future well-designed, large, high-quality research studies are required to validate these findings. This research will likely need to isolate cases where VR is successful based on age cohorts, the type of procedure, and the type and dosage of VR used. In addition, the distinction between state anxiety and pain perception must be clarified and the interconnectedness of the two states investigated as part of the research, along with other emotional states. The content of the VR application also makes a difference on efficacy, for example, game-based VR was more effective in managing pain in children, while adults preferred relaxing environments (6). Content should thus also be taken into account (82).

## Discussion and Future Research Opportunities

Overall, VR has been found to be effective as a complementary form of pain management. The significant findings discussed in this review illustrate the benefits of VR to manage pain. VR is indicated for use in severe clinical cases and also in less severe instances if the cost-benefit is demonstrated (5). The extent to which pharmacological analgesia may be reduced, and opioids substituted or decreased, has significant clinical benefits for physicians and patients (23).

While the majority of results have been positive, it is noted that many studies have small sample sizes, are case studies and do not control for extraneous variables such as the interrelationship between pain and anxiety, and the dosage and type of VR technology used. There are also mixed results for the use of VR to treat chronic pain, and to treat both acute and chronic pain in pediatric populations. There is a necessity to conduct larger RCTs to better understand the use cases for which VR is most effective. Mallari (63) recommends that studies evaluate appropriate dosage of VR exposure compared to other pain analgesia therapies (i.e. pain medications, visual illusions, mirror box therapy, etc.) for both short and long-term outcomes. There is also scope to examine the effects of VR on pain control in different clinical settings (including intensive care, acute care and outpatient settings) (63).

The cost of VR has resulted in the majority of studies being conducted in clinical settings. As the price of technology decreases and as VR becomes more ubiquitous, the potential to include the technology in home-based care in the longer term should be investigated (6, 32). VR as a complementary intervention needs to be available outside of clinical settings and should have real world generalizability and relevance (81).

The number of studies on VR and pain illustrates the interest, efficacy and potential of this area of development for pain management and treatment (20). The efficacy of VR is likely to increase as technology advances and quality improves, and as the effectiveness of certain immersive experiences are compared to one another (81). It also seems pertinent for this technology to be paired with other technology-based interventions such as AI, biofeedback and hypnosis to determine if there would be a compounding positive impact that lasts in the longer term (82).

As our understanding of VR as a complementary form of pain management improves, the use of VR is likely to proliferate. This is an exciting area of inquiry and promises to yield multiple applications.

## Author Contributions

Both authors listed have made a substantial, direct, and intellectual contribution to the work and approved it for publication.

## Conflict of Interest

The authors declare that the research was conducted in the absence of any commercial or financial relationships that could be construed as a potential conflict of interest.

## Publisher's Note

All claims expressed in this article are solely those of the authors and do not necessarily represent those of their affiliated organizations, or those of the publisher, the editors and the reviewers. Any product that may be evaluated in this article, or claim that may be made by its manufacturer, is not guaranteed or endorsed by the publisher.
